# Electronic Structure of Trions in Layered Hybrid Lead Halide Perovskites

**DOI:** 10.1021/acs.jpcc.4c04617

**Published:** 2024-10-08

**Authors:** Juan I. Climente, José L. Movilla, Josep Planelles

**Affiliations:** † Departament de Química Física i Analítica, 16748 Universitat Jaume I, E-12080 Castelló de la Plana, Spain; ‡ Departament d’Educació i Didàctiques Específiques, 16748 Universitat Jaume I, 12080 Castelló, Spain

## Abstract

A theoretical description of trions in layered hybrid organic–inorganic lead halide perovskites is presented. The description is based on a variational effective mass model, including polaronic effects by means of a Haken potential, spatial and dielectric confinements. We show that in thin layers, trions are formed by an exciton plus a more distant, orbiting carrier. Because lattice polarization in these materials is weak at short distances but strong at long ones, trion attractions are less screened than repulsions. The result are binding energies (shift between trion and exciton spectroscopic bands) as large as 70 meV in monolayered structures, greater than those of biexcitons. The ionic character of the bond (uneven sharing of the attractive carrier by the two repelling ones) reaches 50%, stimulated not only by the position-dependent dielectric constant, but also by dielectric confinement, the two effects being nonadditive. A definition of binding energy in confined systems, properly describing the correlated dynamics of particles, is introduced.

## Graphical abstract

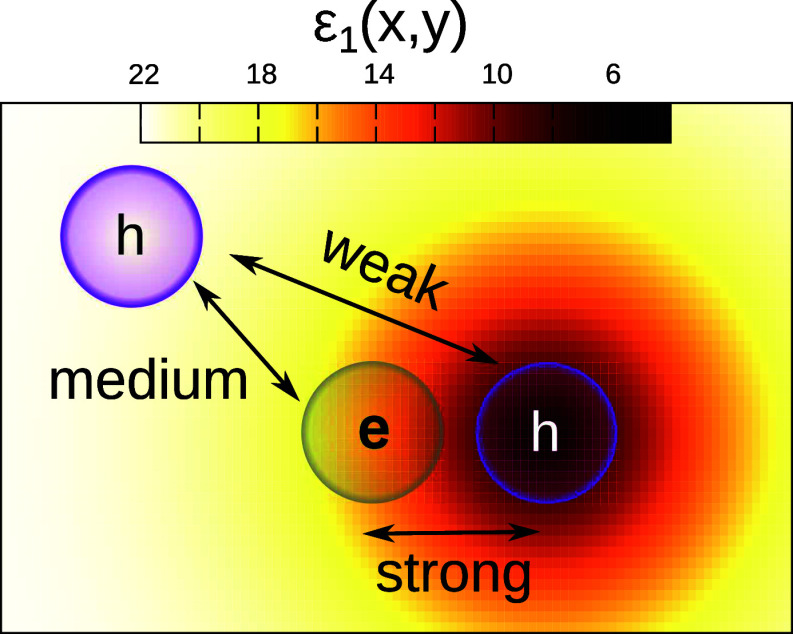

## Introduction

There is currently interest in harnessing the optoelectronic properties of quasi-2D hybrid organic–inorganic lead halide perovskites, such as layered (Ruddlesden–Popper) structures and nanoplatelets. The synergy between default-tolerant optical activity of halide perovskite crystal and the additional functionalities imparted by the organic ligands (chemical stability, quantum confinement, dielectric confinement) turn these layered perovskites into versatile and promising building blocks for practical applications.^1–5^

Precise knowledge on the electronic structure, and its connection with the structural parameters that can be tailored during the synthesis process (layer thickness and composition, dielectric environment) are a requisite to configure and optimize the optical response. The majority of efforts aiming at the elucidation of the electronic structure (binding energy, charge distribution, fine structure) have focused on the study of neutral excitons,^4–13^ and biexcitons,^13,14^ which can be easily photoinduced in experiments.^8,10–12,15,16^ Much less attention has been paid to trions (singly charged excitons) so far.

Trions have intrinsic interest because they combine the strong light-matter coupling of excitons with the electrical charge of a free carrier, which enables simultaneous control of optics and transport.^17,18^ They form spontaneously in colloidal nanoplatelets made of cadmium chalcogenides, where surface defects are prone to trapping holes from excitons. When the next exciton is photogenerated, it binds with the remaining electron to form a negative trion.^19–21^ This has stimulated studies on the electronic properties of such quasi-particles.^22,23^ Spontaneous trions have been reported in lead halide perovskite nanocrystals as well,^24–26^ but their presence in quasi-2D layered structures is more elusive. Recently, Ziegler and co-workers succeeded in intentionally introducing trions (both positive and negative) in thin lead halide layers (PEA_2_PbI_4_), by integrating these structures between graphene contacts.^27^ Binding energies between 30 and 46 meV were observed, depending on the dielectric environment. These values are consistent with earlier predictions from effective mass-diffusion Quantum Monte Carlo simulations.^13^

In the present study, we elaborate further on the electronic structure of trions in layered lead halide perovskites (LHPs). We consider a prototypical Ruddlesden–Popper perovskite, namely (PEA)_2_(MA)_*n*−1_Pb_*n*_I_3*n*+1_, with *n* the number of sublayers, PEA = phenylethylammonium and MA = methlyammonium. The trion geometry, bond nature and binding energy are analyzed as a function of the structure thickness (*n*) and dielectric environment. We do so by resorting to an effective mass - variational Quantum Monte Carlo model. Analogous models have been used by us to investigate excitons^9^ and biexcitons^14^ in these systems, obtaining quantitative agreement with experimental measurements of binding energies.

Our results extend earlier theoretical studies^13^ by including the influence of the different polarizabilities of the lattice at short and long distances from a source carrier.^28,29^ This effect has proved important in LHPs because the strong lattice polarizability implies very different dielectric constants at short distances (where electronic screening prevails, ϵ_*∞*_) and long ones (where ionic screening adds up, ϵ_s_).^9,10,14,30,31^ We shall see that in a positive trion (two holes, one electron), the ground state charge distribution is such that one of the holes stays close to the electron, while the other stays relatively far (see Table of Contents graphic). The former couple benefits from the weak screening at short distances (ϵ → ϵ_*∞*_), which maximizes the attraction. The second hole, on the other hand, is far enough from the first hole for repulsions to be governed by ϵ → ϵ_s_, which reduces repulsions. All in all, this translates into very large trion binding energies (up to 70 meV for exfoliated layers with *n* = 1, when defined as the energy difference between trion and excitonic bands), a mixed covalent-ionic character of the trion bond (uneven sharing of the attractive carrier by the two repelling ones) and large correlation energies.

## Theoretical Method

We calculate the ground state energy and wave function of a positive trion, although the procedure is analogous for the negative one. The Hamiltonian is based on a *k* · *p* Hamiltonian for two uncoupled (conduction and valence) bands:

$$H_{X^{+}}=\underset{i=e,h_{1},h_{2}}{\sum }\left(\frac{{\mathrm{p̂}}^{2}}{2m_{i}}+V_{i}\right)+V_{c}\left(r_{h_{1}},r_{h_{2}}\right)+\underset{i=h_{1},h_{2}}{\sum }V_{c}\left(r_{e},r_{i}\right)+E_{gap}$$ 1

Here, *m* is the effective mass, **p̂** the momentum operator, and *E*_gap_ is the bulk band gap of the perovskite crystal. The single particle potential is *V*_*i*_ = *V*_*i*_^conf^ + *V*_*i*_^self^, where *V*_*i*_^conf^ is the spatial confining potential. In our model, we describe the layered perovskite as a cuboid with large lateral dimensions (*L*_*x*_, *L*_*y*_ ≫ *L*_*z*_, such that lateral confinement is negligible–see schematic in Figure 1a). *V*_*i*_^conf^ = 0 inside the cuboid and infinite outside it. *V*_*i*_^self^ is the self-energy potential. *V*_*c*_(**r**_*i*_, **r**_*j*_) terms represent the Coulomb interaction between carriers. Both *V*_*i*_^self^ and *V*_*c*_(**r**_*i*_, **r**_*j*_) account for dielectric confinement by using quantum well image charges, with inclusion of long- and short-range interactions. Detailed expressions are given in ref 9. They are based on Haken potential,^28^ a simple model of exciton-polaron interactions, which has been shown to improve estimates of exciton and biexciton binding energies in LHPs.^9,10,14,30,31^

**Figure 1:**
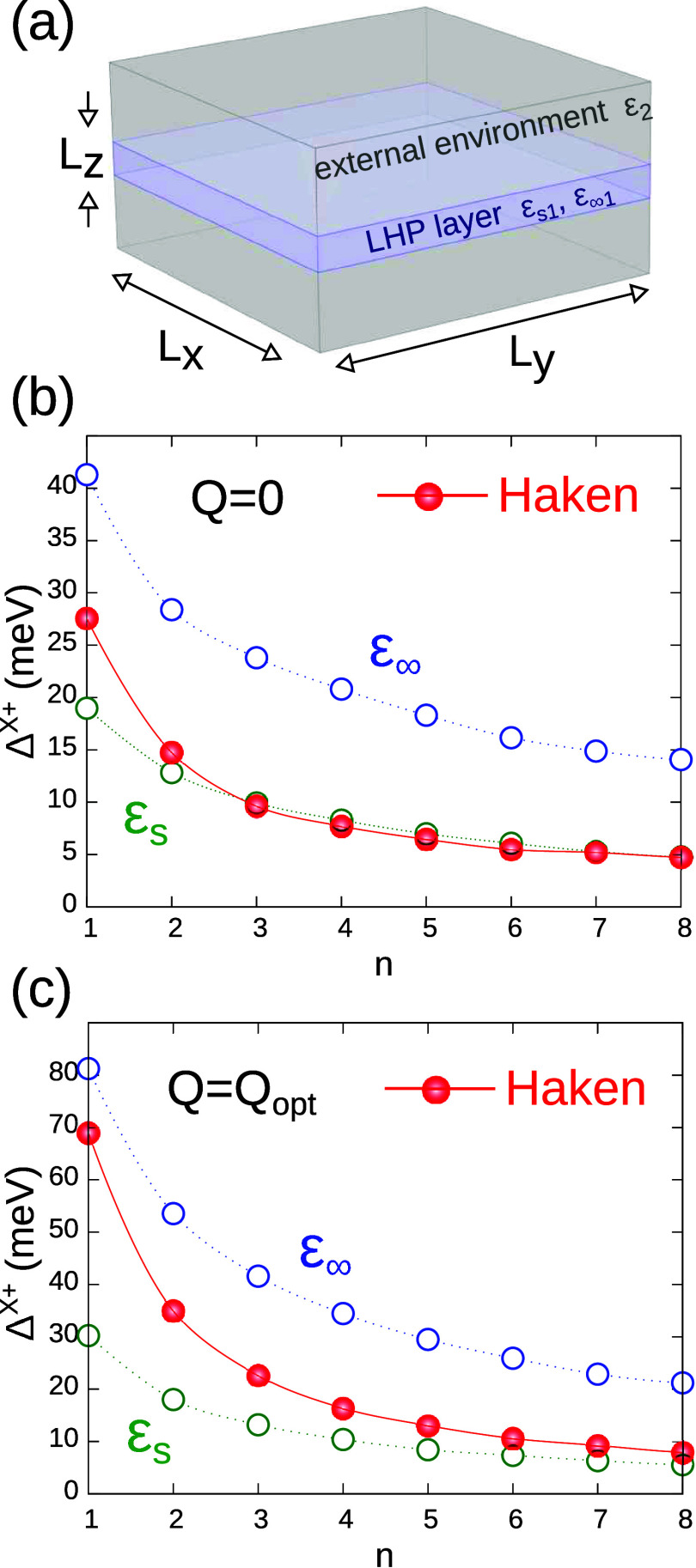
(a) Schematic of the system under study. We consider *L*_*x*_, *L*_*y*_ ≫ *L*_*z*_ (negligible lateral confinement). (b) Trion binding energy as a function of the number of sublayers, for a pure covalent bond (*Z*_1_ = *Z*_2_). (c) Same but including ionic contributions to the bond (*Z*_1_ ≠ *Z*_2_). In (b) and (c), red dots show the energies obtained using Haken’s potential, green dots those using a Coulomb potential with static dielectric constant, and blue ones those with a dynamic constant. Lines are guide to the eyes. Ionic contributions greatly enhance binding energies.

To assist with the interpretation of the results, it is worth introducing here the basic Haken potential (for bulk systems, prior to inclusion of quantum and dielectric confinement). In atomic units, the potential exerted by a source charge *i* on a test charge *j* reads^28^:

$$V_{ij}^{\mathrm{bulk}}(r)=\frac{q_{i}q_{j}}{\epsilon_{s}r}+\frac{q_{i}q_{j}}{\epsilon^{*}r}\frac{e^{-\beta_{i}r}+e^{-\beta_{j}r}}{2}$$ 2

Here, *r* is the distance between charges, *q* the elementary charge (positive or negative) and ϵ_s_ the static dielectric constant. The term $\left(\frac{1}{\epsilon_{\infty }}-\frac{1}{\epsilon_{s}}\right)=\frac{1}{\epsilon^{*}}$ represents the ionic screening of carriers, with ϵ_*∞*_ the high frequency dielectric constant and β_*i*_ the inverse of the electron or hole polaron radius, *l*_*i*_, which is related to the longitudinal optical phonon frequency. One should note that the first term in eq 2 is a Coulomb interaction with full dielectric screening (electronic plus ionic, ϵ_s_), which prevails at long distances, *r* ≫ *l*_*i*_. On the other hand, the second term is a short-range (Yukawa) one, which starts prevailing at distances *r* ≲ *l*_*i*_, where the ionic contribution to dielectric screening is lost (ϵ_*∞*_ dominates).

To integrate Hamiltonian ([Disp-formula eq1]), we rely on a variational Quantum Monte Carlo method.^32,33^ The variational energy reads:

$$\left\langle E\rangle =\frac{\int dR\Psi^{*}(R)H_{X^{+}}\Psi (R)}{\int dR^{\prime }\Psi^{*}(R^{\prime })\Psi (R^{\prime })}=\int dR\,\;\;p(R)E_{L}(R\right)$$ 3

where Ψ­(**R**) is the trial function, with **R** = (**r**_*e*_, **r**_*h*1_, **r**_*h*2_), *E*_*L*_ the local energy:

$$E_{L}\left(R\right)=\frac{{Ĥ}_{X^{+}}\Psi \left(R\right)}{\Psi \left(R\right)}$$ 4

and *p*(**R**) the probability distribution:

$$p\left(R\right)={\left|\Psi \left(R\right)\right|}^{2}/\int dR^{\prime }{\left|\Psi \left(R^{\prime }\right)\right|}^{2}$$ 5

The Metropolis algorithm is used for importance sampling.^34^ Thus, particles are moved to different trial positions in a random walk. The new positions are accepted if the following condition is satisfied:

$$\frac{p(R_{\mathrm{new}})}{p(R_{\mathrm{old}})}>w$$ 6

where *w* is a random number homogeneously distributed between 0 and 1. For a sufficiently long calculation, this random walk allows to simplify eq 3 as

$$\left\langle E\right\rangle \approx \frac{1}{N}\overset{N}{\underset{i=1}{\sum }}E_{L}\left(R_{i}\right)$$ 7

where *N* is the number of (accepted) points *R*_*i*_ taken in the walk. An improved energy estimator has been proposed, which yields the same average but with smaller variance, by considering not only accepted steps, but also rejected ones^32^:

$$\left\langle E\rangle \approx \frac{1}{N}\sum_{i=1}^{N}[p(R_{\mathrm{new}})E_{L}(R_{\mathrm{new}})+(1-p(R_{\mathrm{new}}))E_{L}(R_{\mathrm{old}})\right]$$ 8

with *N* here being the total number of attempted moves (whether accepted or not). We estimate the energy with eq 8 in our codes. For details (how the local energy is evaluated, the use of random walkers distributed among different CPU processes using OpenMP parallelization, and computational codes) see ref 35. The specific routines of local kinetic and potential energy for the variational function of the present work (see next section) are provided in the Supporting Information.

### Trion Variational Wave Function

In previous papers dealing with CdSe nanoplatelets, we used the following Slater-Jastrow trial wave function for the doublet ground state^22,35^ :

$$\Psi_{X^{+}}(r_{e},r_{h1},r_{h2},\sigma_{e},\sigma_{h1},\sigma_{h2})=\Phi_{e}(r_{e})\Phi_{h}(r_{h1},r_{h2})J(r_{1},r_{2},r_{12})\sigma_{e}[\alpha_{h1}\beta_{h2}-\beta_{h1}\alpha_{h2}]$$ 9

with Φ_*e*_(**r**_*e*_) Φ_*h*_(**r**_*h*1_, **r**_*h*2_) the analytical independent particle wave function, σ_*e*_ [α_*h*1_ β_*h*2_ – β_*h*1_ α_*h*2_] the spin function and *J*(*r*_1_, *r*_2_, *r*_12_) the following Jastrow factor:

$$J(r_{1},r_{2},r_{12})=e^{-Zr_{1}}e^{-Zr_{2}}e^{br_{12}/(1+ar_{12})}$$ 10

where *r*_1(2)_ = |**r**_*e*_ – **r**_*h*1(2)_| and *r*_12_ = |**r**_*h*1_ – **r**_*h*2_|. The first two terms are short-range cusp forms describing the correlation of each hole with the electron, first successfully introduced by Hylleraas^36,37^ on the He atom ground state calculation. *Z* = ζ/*r*_*B*_^*X*^ is a variational coefficient, with ζ the parameter to be varied and *r*_*B*_^*X*^ the (mass and dielectric-constant dependent) Bohr radius. The last term is a Padé Jastrow factor, which has the property of giving the desired limits with *r*_12_. At short ranges of interaction, *r*_12_ → 0, the term becomes e^*b* *r*_12_^, which provides a cusp to compensate for the divergence in hole–hole Coulomb repulsion (*b* > 0). At the same time, the probability to find distant holes (*r*_12_ → *∞*) is more likely than that of proximal holes (*r*_12_ → 0) by a factor (e^*b*/*a*^)^2^. We define *b* = β/*r*_*B*_^*X*^ and *a* = α/*r*_*B*_^*X*^, and let β and α be the variational parameters. This Jastrow factor, originally proposed by Reynolds et al.,^38^ has been shown to be nearly exact for He.^39^ It has later been employed in the ground state variational wave function of the He atom.^40^ An approximate linear expansion of this exponential neglecting the *a* parameter, 1 + *b* *r*_12_, has been used in studies of trions in perovskite crystals.^13^ It can be traced back to the factor employed by Chandrasekhar^41^ and the simplest He ground state Hylleraas wave function.^42^

As pointed out above, the (hydrogen wave function-like) exponential describing the correlation of each hole with the electron is well suited for the He atom. However, as pointed out by Chandrasekhar, it fails to describe the hydrogen negative anion *H* ^–^ as a stable structure.^41^ This fact led him to propose a different screening for each electron, i.e., to replace the factor e^–*Z* (*r*_1_+*r*_2_)^ by e^(−*Z*_1_ *r*_1_–*Z*_2_ *r*_2_)^ + e^(−*Z*_1_ *r*_2_–*Z*_2_ *r*_1_)^, the two adding terms coming from the indistinguishability of both electrons. This change is reminiscent of switching from restricted to unrestricted Hartree–Fock methods, which enables a correct description of the *H*_2_ molecule beyond the region of equilibrium interatomic distance, where restricted Hartree–Fock fails.^43^ Then, although it implies introducing an extra variational parameter, the new wave function yields an improved description of the electronic structure of *H* ^–^ systems, where interparticle distances are longer than in He. As in the comparison of unrestricted and restricted Hartree–Fock, the two-parameter wave function (with *Z*_1_ and *Z*_2_) renders a better description for longer interparticle distances, while preserving the quality of the single-parameter one at short distances. In particular, Rioux reports that the deviation of energy with this optimized trial wave function for He is within 1% of the actual ground state energy of He.^44^

In this study we are interested in trions. Negative and positive trions can be related to the well-known atomic systems of *H* ^–^ and *H*_2_^+^. Unlike in atoms, however, the mass ratio of charges in trions, *m*_*e*_/*m*_*h*_, varies in a wide range. Thus, the negative *X* ^–^ and positive *X* ^+^ trion complexes can qualitatively change in their structures and properties from the positronium ion in one limit (*m*_*e*_ ≈ *m*_*h*_) to *H* ^–^ or *H*_2_^+^ in the other limit (*m*_*e*_ ≪ *m*_*h*_). In the case of LHPs, where *m*_*e*_ ≈ *m*_*h*_, we are close to the positronium limit, and the Born–Oppenheimer approximation does not hold. A wave function behaving evenly for any *m*_*e*_/*m*_*h*_ is then needed. Hill^45,46^ showed that Chandrasekhar’s wave function produces binding of the same quality for any mass ratio.

For all the above reasons, in the present study we change the Jastrow factor from eq 10 (restricted screening) to Chandrasekhar’s form (unrestricted screening). Specifically, we define $Z_{1}=\frac{Z}{2}\left(1+Q\right)$ , $Z_{2}=\frac{Z}{2}\left(1-Q\right)$ , *s* = *r*_1_ + *r*_2_ and *t* = *r*_1_ – *r*_2_. The electron–hole correlation factor is now given by

$$e^{\left(-Z_{1}r_{1}-Z_{2}r_{2}\right)}+e^{\left(-Z_{1}r_{2}-Z_{2}r_{1}\right)}=2e^{-Zs/2}\cos h[ZQt/2]$$ 11

and the hole–hole correlation factor by $e^{Z\beta r_{12}/(1+Z\alpha r_{12})}$ . Then, the complete (unnormalized) trial wave function–omitting spin degrees of freedom– reads:

$$\Psi_{X^{+}}=\Phi_{e}(r_{e})\Phi_{h}(r_{h1},r_{h2})e^{-Z(r_{1}+r_{2})/2}\cos h[ZQ(r_{1}-r_{2})/2]e^{Zbr_{12}/(1+Zar_{12})}$$ 12

As in ref 35, *Z* = ζ/*r*_*B*_^*X*^ is a variational coefficient, with ζ the parameter to be varied and *r*_*B*_^*X*^ the mass and dielectric constant dependent Bohr radius, while the rest of parameters (*Q*, *a*, *b*) are not scaled with *r*_*B*_^*X*^.

More sophisticated trial functions have been suggested for trions, with additional variational parameters often aiming at providing a more accurate description of repulsions.^47^ The present proposal has the advantage of keeping the smallest number of parameters that captures the correct limit behavior, while being physically consistent with that of neutral excitons.^9,35^ Also, in all our calculations the energy dependence on the repulsive correlation factor is minor, which explains why the linear expansion used in refs 13,41,42 works well.

### Trion Geometry Determination

On average, the permutation symmetry in Ψ_*X* ^+^_ makes the electron–hole distances equal for both holes, ⟨*r*_1_⟩ = ⟨*r*_2_⟩. However, we can discriminate the different behavior of the two holes by calculating, in every step of the Metropolis random walk, which hole is closer and farther to the electron, and hence determining average values ⟨*r*_*m*_⟩ and ⟨*r*_*M*_⟩ instead. We follow the next procedure for assigning the geometry to a system composed of two indistinguishable holes and an electron, all of which are of similar mass. On the one hand, the hole–hole *r*_12_ distance is well-defined. However, as stated above, it is not the case for the distances *r*_1_, *r*_2_ between each hole and the electron, due to the indistinguishability of both holes. In this case it only makes sense referring to the longest and shortest distance between the electron and a hole, *r*_*M*_ and *r*_*m*_, respectively. Then, to determine the trion distances we proceed as follows: after thermalization, along the random walk, we determine the Cartesian coordinates of the three particles as the required input to calculate the associated local energy. From them, we calculate the distances *r*_12_, *r*_*m*_ = min­(*r*_1_, *r*_2_) and *r*_*M*_ = max­(*r*_1_, *r*_2_). This introduces some bias, because even in symmetric structures with independent electrons and holes, at every step of the walk there is one hole closer and another farther away from the electron. We reduce the bias statistically. Along the random walk, when calculating *r*_*m*_ = min­(*r*_1_, *r*_2_) and *r*_*M*_ = max­(*r*_1_, *r*_2_) from a given set of Cartesian coordinates, we define the ratio τ = *r*_*m*_/*r*_*M*_. By definition, 0 ≤ τ ≤ 1. Then, we call for a random number *w* and claim that if τ > *w* our system is a set of independent particles and then we set *r*_*m*_ = *r*_*M*_ = (*r*_1_ + *r*_2_)/2. Otherwise, *r*_*m*_ = min­(*r*_1_, *r*_2_), *r*_*M*_ = max­(*r*_1_, *r*_2_).

In addition, we introduce a bias-free control parameter, *k* = (⟨*r*_*m*_⟩ + ⟨*r*_*M*_⟩)/(2⟨*r*_12_⟩). Should the three confined particles be independent, the distances between any two particles must be the same as between any other two i.e., it must happen that ⟨*r*_12_⟩ = ⟨*r*_1_⟩ = ⟨*r*_2_⟩ (that is, the trion forms an equilateral triangle). Along the procedure, the averaged shorter ⟨*r*_*m*_⟩ is necessarily smaller than the averaged longer ⟨*r*_*M*_⟩. All the same, the hole–hole ⟨*r*_12_ ⟩ distance is unambiguously determined. Because the procedure is linear, the average (⟨*r*_*m*_⟩ + ⟨*r*_*M*_⟩)/2 must equal ⟨*r*_12_⟩. We have checked that this is indeed the case in all our calculations of (*Z*, *Q*, β, α) = (0, 0, 0, 0), for which *k* = 1. By contrast, for the optimal variational parameters, *k* < 1 is obtained, which confirms a departure from the equilateral triangle geometry.

## Results and Discussion

We study positive trions confined within a layer of (PEA)_2_(MA)_*n*−1_Pb_*n*_I_3*n*+1_, with *n* the number of sublayers (PbI_4_ octahedra). Dielectric constants inside the perovskite layer are ϵ_s1_ = 22.0 and ϵ_*∞* 1_ = 5.6,^48^ effective masses *m*_*e*_ = 0.19 and *m*_*h*_ = 0.22 and the polaron radii of *l*_*e*_ = 1/β_*e*_ = 0.94 nm and *l*_*h*_ = 1/β_*h*_ = 1.01 nm. These values successfully reproduced the experimental exciton binding energies of (PEA)_2_(MA)_*n*−1_PbnI_3*n*+1_ layers, with *n* = 1, 2, 3.^9^ For simplicity, we disregard the mass dependence on *n*, which is moderate.^11^ Also, consecutive layers are considered to be far enough for the composite picture of independent layers to hold.^5,15^ The interaction with neighbor layers is then captured by a modulation of the (averaged) dielectric constant of the environment. The low polarizability of the organic environment is characterized by ϵ_2_ = 2,^8^ and this value increases in the presence of nearby inorganic layers.^4,11^ In our study we will fix ϵ_2_ = 2, so that we obtain upperbound estimates on the influence of dielectric confinement. The lateral dimensions of the system are set to *L*_*x*_ = *L*_*y*_ = 30 nm, see Figure 1a. This is close to the regime of no lateral confinement, expected for quasi-2D layered perovskites. The layer thickness is *L*_*z*_ = *n a*, with *a* = 0.63 nm the lattice constant.^49^ We stress that, using the same approximations and material parameters, quantitative agreement with the experimental binding energies of neutral excitons (with *n* = 1, 2, 3) and biexcitons (with *n* = 1) is achieved.^9,14^

### Influence of Distance-Dependent Dielectric Screening

We start by analyzing the effect of quantum confinement on the trion binding energy. The binding energy is defined here as Δ^*X* ^+^^ = *E* ^*X*^ + *E* ^*h*^ – *E* ^*X* ^+^^, where *E* ^*X*^, *E* ^*h*^ and *E* ^*X* ^+^^ are the energies of the exciton, free hole, and trion ground states in the nanostructure. This value closely corresponds to the spectroscopic shift between excitons and trions. We will compare results where we set *Q* = 0 and *Q* = 1 in the variational wave function, eq 12. Setting *Q* = 0 implies using a three-parameter function, where both holes share the electron equally (*Z*_1_ = *Z*_2_ = *Z*). We shall refer to this as the covalent limit. Using this approximation, close agreement with experimental trion spectra was found in CdSe nanoplatelets.^22^ Unlike in CdSe, however, the mass ratio of halide perovskites is *m*_*e*_/*m*_*h*_ ≈ 1, and the validity of the approximation is not granted. Enabling *Q* > 0, on the other hand, permits asymmetric sharing of the electron by the two holes (*Z*_1_ ≠ *Z*_2_). We shall refer to this as the ionic contribution to the bond. As mentioned before, the extra stabilization provided by *Q* > 0 is reminiscent of that provided by unrestricted Hartree–Fock (with open-shell configurations) over restricted Hartree–Fock (closed-shell configuration only) in the *H*_2_ molecule.

In Figure 1b, red dots show Δ^*X* ^+^^ calculated with *Q* = 0 and full inclusion of effects (quantum confinement, dielectric confinement, Haken potential). The figure shows that the binding energy increases as the layer thickness (*n*) decreases, which is the expected consequence of evolving toward a 2D system.^13^ The highest value is obtained for a single sublayer (*n* = 1, PEA_2_PbI_4_ structure), where Δ^*X* ^+^^ = 27.5 meV. This number grossly underestimates the large binding energies recorded in experiments for such systems (up to 46 meV).^27^ Another unexpected issue in Figure 1b is that Δ^*X* ^+^^ is close to the static limit (green dots in the figure), where the interaction between carriers is modeled as a standard Coulomb term with distance-independent static screening, ϵ_1_ = ϵ_s1_. This occurs for all but the thinnest structures (*n* = 1). Even then, Δ^*X* ^+^^ remains far from the dynamic limit (Coulomb term with ϵ_1_ = ϵ_*∞*1_, blue dots). This is at odds with the trend observed for excitons and biexcitons, where binding energies evolve from one limit to another, reaching values close to the dynamic one for *n* = 1.^9,14^ As a matter of fact, this issue explains the lack of agreement with experimental binding energies, which suggest an effective (distance-averaged) dielectric constant ϵ_1_ = 6.1, far from the static limit observed here (ϵ_1_ ≈ 22).

In Figure 1c, we recalculate the trion binding energies by introducing *Q* as a variational parameter. The changes are substantial. First, the simulations using Haken’s potential (red dots) now succeed in retrieving the same behavior as observed for excitons and biexcitons, by evolving from the ϵ_s_ limit to the proximity of the ϵ_*∞*_ one as *n* decreases. Second, values as large as Δ^*X* ^+^^ = 70 meV are reached when *n* = 1. These binding energies are well above those calculated^13,14^ and measured^12,16,50,51^ for biexcitons in the same structure (Δ^*XX*^ = 40–55 meV). This result suggests that, under conditions of strong quantum and dielectric confinement, the spectral bands of trions and biexcitons in layered perovskites are susceptible of reversing with respect to usual order in 3D nanocrystals,^52^ as is actually the case in transition metal dichalcogenides.^53,54^ The experimental values of Δ^*X* ^+^^ = 30–48 meV reported in ref 27 are likely indicative that the dielectric constant of the environment was larger than ϵ_2_ = 2.

A note is due on the accuracy of the binding energies we calculate. The accuracy is certainly subject to (i) the fitness of the material parameters we use, and (ii) to the capability of our variational wave function to capture electronic correlations. With regard to (i), the agreement of our earlier simulations with experimental exciton and biexciton binding energies in thin LHPs^9,14^ seems to support the choice of parameters. As for (ii), because we use exact (or nearly so) exciton and hole eigenstates, if the variational calculation misses trion correlation energy, the binding energy we calculate must be a lower bound of the experimental one.

The results in this section can be rationalized as follows. With decreasing layer thickness (*n*), the enhanced quantum and dielectric confinement tend to reduce electron–hole distances. This effect is further magnified by short-term (Yukawa) interactions. Yet, the covalent model (*Q* = 0, rigid wave function) fails to retrieve its dynamic limit because squeezing all particles under the polaronic radius (where ε → ε_*∞*_) implies large confinement energies. By contrast, when ionicity is introduced (*Q* > 0), the wave function is more flexible. Quantum confinement is relaxed by placing one carrier farther from the exciton and still benefit from enhanced attraction and reduced repulsion.

### Influence of Bond Ionicity

The substantial changes between Figure 1b, where the trion bond is purely covalent (*Z*_1_ = *Z*_2_) and Figure 1c, where ionic contributions are introduced (*Z*_1_ ≠ *Z*_2_), imply that the latter play a major role in layered hybrid LHPs. To gain direct insight into this point, we calculate Δ*E*_*Q*_ = *E* ^*X*+^(*Q*_opt_) – *E* ^*X* ^+^^(*Q* = 0). Here, *E* ^*X* ^+^^(*Q* = 0) is the energy of the three-parameter wave function, Ψ_*X* ^+^_, with optimized values (*Z*_opt_, β_opt_, α_opt_)_*Q*=0_. *E* ^*X* ^+^^(*Q*_opt_) is the energy of the four-parameter function, where the same (*Z*, β, α) parameters are held, but *Q* is optimized. Figure 2a shows Δ*E*_*Q*_ as a function of the layer thickness. One can see that the relevance of the ionic correction increases with quantum confinement. For *n* = 1 (PEA_2_PbI_4_ layer), Δ*E*_*Q*_ reaches −40 meV. This stabilization accounts for the large enhancement of Δ^*X* ^+^^ observed in Figure 1c as compared to Figure 1b.

**Figure 2:**
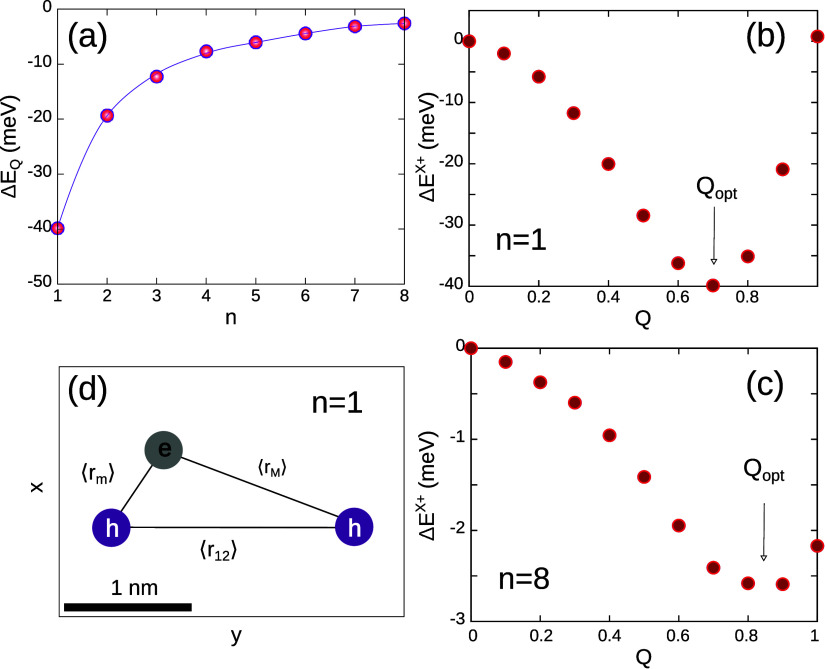
(a) Trion energy stabilization when ionic contributions are added to the covalent bond. The thinner the layered perovskite, the more important they are (b, c) trion energy as a function of the variational parameter *Q*. A minimum is found at intermediate values between the covalent limit (*Q* = 0) and the dissociated trion limit (*Q* = 1). The minimum is deeper for thin (*n* = 1) layers than for thick (*n* = 8) ones. In both panels the reference energy is that of the *Q* = 0 ground state. (d) Most probable charge distributions in a PEA_2_PbI_4_ layer. Notice the asymmetric sharing of the electron by the two holes.

Figure 2b,c shows the trion energy as a function of the variational parameter *Q*, for thin (*n* = 1) and thick (*n* = 8) LHP layers, respectively. The rest of variational parameters are fixed at the optimal values of the three-parameter function, (*Z*_opt_, β_opt_, α_opt_)_*Q*=0_. As mentioned before, *Q* = 0 represents the fully covalent limit. Therefore, the deviation from this limit quantifies the ionic contribution to the trion bond energy. On the other hand, at *Q* = 1 the electron–hole correlation factor – eq 11– becomes e^–*Zr*_1_^ + e^–*Zr*_2_^, which implies that the electron correlates either with one hole or with the other. This is qualitatively related with the trion dissociation limit (we will elaborate on this point in the next section). As can be seen in the figures, in all cases a minimum is found at *Q*_opt_ in between the two boundaries, *Q* = 0 and *Q* = 1. The minimum of *Q*_opt_ is deeper for the thin LHP layer, which implies that quantum and dielectric confinement stimulate the ionic character of the bond and prevent trion dissociation.

The mixed covalent-ionic chacter of the trion bond is reflected in its geometry as well. As described in the geometry determination subsection, on average, the permutation symmetry in Ψ_*X* ^+^_ makes the electron–hole distances equal for both holes, ⟨*r*_1_⟩ = ⟨*r*_2_⟩. However, we discriminate the different behavior of the two holes by calculating, in every step of the Metropolis random walk, which hole is closer and farther to the electron, and hence determining average values ⟨*r*_*m*_⟩ and ⟨*r*_*M*_⟩ instead. From ⟨*r*_*m*_⟩, ⟨*r*_*M*_⟩ and *r*_12_ – the hole–hole distance–, we derive the trion geometries of a thin (*n* = 1) LHP layer, which are shown in Figure 2d. The figure reveals that the trion is formed by an electron–hole pair (exciton) and a more distant hole, in line with the increased ionic character we anticipated from the binding energies.

We have observed in Figure 2a that the ionic stabilization term Δ*E*_*Q*_ becomes increasingly important for thin LHP layers. This may be due to the stronger quantum confinement, the stronger dielectric confinement, and/or the increasingly important role of distance-dependent polaronic interactions. To disentangle these factors, in Figure 3 Δ*E*_*Q*_ is calculated with a gradual inclusion of the corresponding terms in the Hamiltonian. Orange dots show the stabilization when both dielectric confinement and the short-range term of the Haken potential are turned off (we label this as (*d*, *y*) = (0, 0), where *d* stands for dielectric confinement and *y* for the Yukawa term in eq 2). Only quantum confinement is present, with bulk-like Coulomb interactions screened by ϵ_*s*1_. In this case, the ionic stabilization is marginal. Activating either dielectric confinement (green dots, (*d*, *y*) = (1, 0)) or the short-range interactions (gray dots, (*d*, *y*) = (0, 1)) leads to significant enhancement of Δ*E*_*Q*_, especially for small *n*. Interestingly, short-range interactions have a greater influence than dielectric confinement. This is because separating one hole in the presence of distance-dependent screening is an efficient mechanism of stabilization: the proximate electron–hole pair benefits from the weak short-range screening, while the distant holes experience strong long-range screening. Last, considering all the terms altogether ((*d*, *y*) = (1, 1), red dots) leads to the large stabilization anticipated in Figure 2a. It is worth noting that the contribution of dielectric and distance-dependent interactions is nonadditive. The net stabilization is greater than the sum of the two individual contributions, which shows that they have a synergistic interaction.

**Figure 3:**
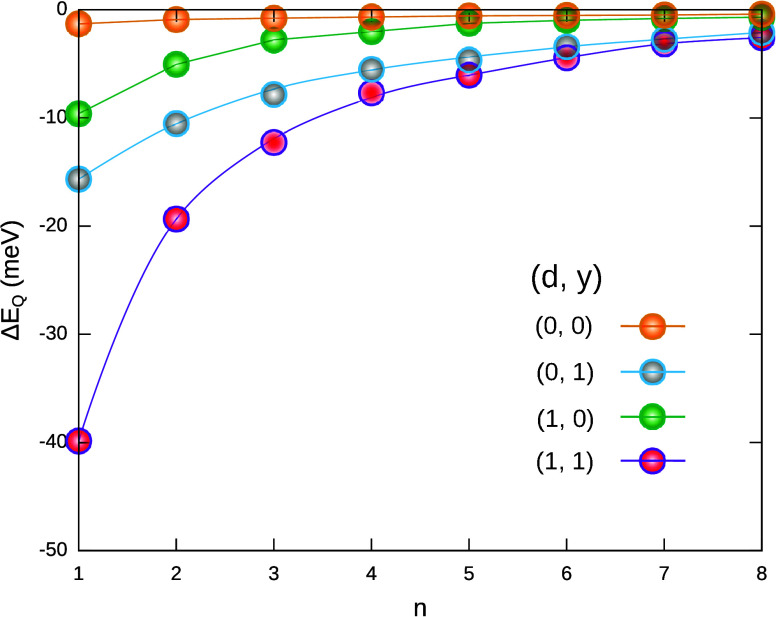
Trion energy stabilization upon activation of ionic contributions, as a function of the LHP thickness. The stabilization is calculated in the presence of different physical factors. *d* = 0 (*d* = 1) stands for absent (present) dielectric confinement. *y* = 0 (*y* = 1) stands for absent (present) short-range interactions in Haken’s potential.

### Binding Energy

The binding energy is a key parameter that governs the physics of many optoelectronic devices.^55^ In confined systems, however, the definition of trion binding energy is ambiguous. In the literature, it is customary to express it as we did in earlier sections. Namely,

$$\Delta^{X^{+}}=E^{X}+E^{h}-E^{X^{+}}$$ 13

where *E* ^*X*^, *E* ^*h*^ and *E* ^*X* ^+^^ are the energies of exciton, hole and trion in the same nanostructure. This is an accurate definition in bulk, where it gives the difference in energy of the dissociation process:

$$X^{+}\to h+X$$ 14

because the resulting hole and exciton are independent, the most likely configuration is that where they are at infinite distance, thus feeling no interaction. Yet, in confined systems, the hole and exciton coexist in a finite region of space and feel each other through Coulomb interaction. Then, *E* ^*X*^ + *E* ^*h*^ is no longer a good estimate of the energy in the right-hand side of eq 14. The reason why the use of Δ^*X* ^+^^ is widespread is because it closely relates to the separation between trion and exciton spectroscopic peaks, which is an accessible experimental magnitude. The energy of the trion optical transition *X* ^+^ → *h* is Δ*E*_*X* ^+^_ = *E*_*h*_ – *E*_*X* ^+^_, while that of the exciton transition *X* → Ø is Δ*E*_*X*_ = 0 – *E*_*X*_. The spectroscopic shift is then Δ*E*_*X* ^+^_ – Δ*E*_*X*_ = *E*_*h*_ + *E*_*X*_ – *E*_*X* ^+^_ = Δ^*X* ^+^^. Hereafter, we refer to Δ^*X* ^+^^ as the spectroscopical binding energy.

A more physically meaningful definition of the binding energy in confined systems should give the energy that prevents the system from dissociating into uncorrelated subsystems. That is, into subsystems whose motion is independent. In the dissociation process of eq 14, the resulting exciton and hole are uncorrelated but, because of the confinement, they still feel Coulomb interaction. The associated binding energy in this system is then:

$$E_{b,2}^{X^{+}}=E^{X+h}-E^{X^{+}}$$ 15

where *X* + *h* stands for an uncorrelated exciton and hole, both simultaneously confined in the same space. Within our model, *E*_*X*+*h*_ can be calculated as *E*(*Z*_opt_, *Q* = 1, β = 0, α = 0), which corresponds to the wave function Ψ_*X* ^+^_ = Φ_*e*_(*r*_*e*_) Φ_*h*_(*r*_1_, *r*_2_) (e^–*Zr*_1_^ + e^–*Zr*_2_^). This definition is related to the dissociation process discussed in Figure 2b,c, albeit in there we calculated the energy for (*Z*_opt_ *′*, *Q* = 1, β_opt_ *′*, α_opt_ *′*)_*Q*=0_, and here we do so for (*Z*_opt_, *Q* = 1, β = 0, α = 0)_*Q*=1_. In other words, before we had frozen the exciton interaction to the covalent limit (*Q* = 0), and in a second step we had suppressed the electron correlation with the extra hole (by setting *Q* = 1). Now, we let the exciton interaction relax to reach the most stable energy regardless of the extra hole (*Q* = 1), and suppress hole–hole correlation. This is a quantitatively valid definition for the lowest dissociated trion state.

Another quantity of interest is the energy required to split the trion into three uncorrelated subsystems,

$$X^{+}\to h_{1}+h_{2}+e$$ 16

The associated binding energy is

$$E_{b,3}^{X^{+}}=E^{e+2h}-E^{X^{+}}$$ 17

Again, here the products of eq 16 are uncorrelated but coexist in a finite region of the space and hence interact via Coulomb terms. Within our model, *E* ^e+2*h*^ can be calculated as *E*(*Z* = 0, *Q* = 0, β = 0, α = 0), which corresponds to the wave function Ψ_*X* ^+^_ = Φ_*e*_(*r*_*e*_) Φ_*h*_(*r*_1_, *r*_2_).

The trion binding energies *E*_*b*,2_ and *E*_*b*,3_ are represented in Figure 4, as a function of the LHP layer thickness. *E*_*b*,2_ ranges from 1 to 16 meV. By contrast, *E*_*b*,3_ is much larger, exceeding 300 meV for *n* = 1 layers. The contrast between the two cases indicates that most of the correlation energy is within the exciton, with a minor contribution coming from the exciton-hole interaction. The fact that *E*_*b*,2_ is much smaller than the spectroscopic binding energy (Δ^*X* ^+^^, cf. Figure 1c), and below thermal energy at room temperature, implies that trions–even when significantly red-shifted with respect to excitons–, will only keep exciton-hole correlation at cryogenic temperatures.

**Figure 4:**
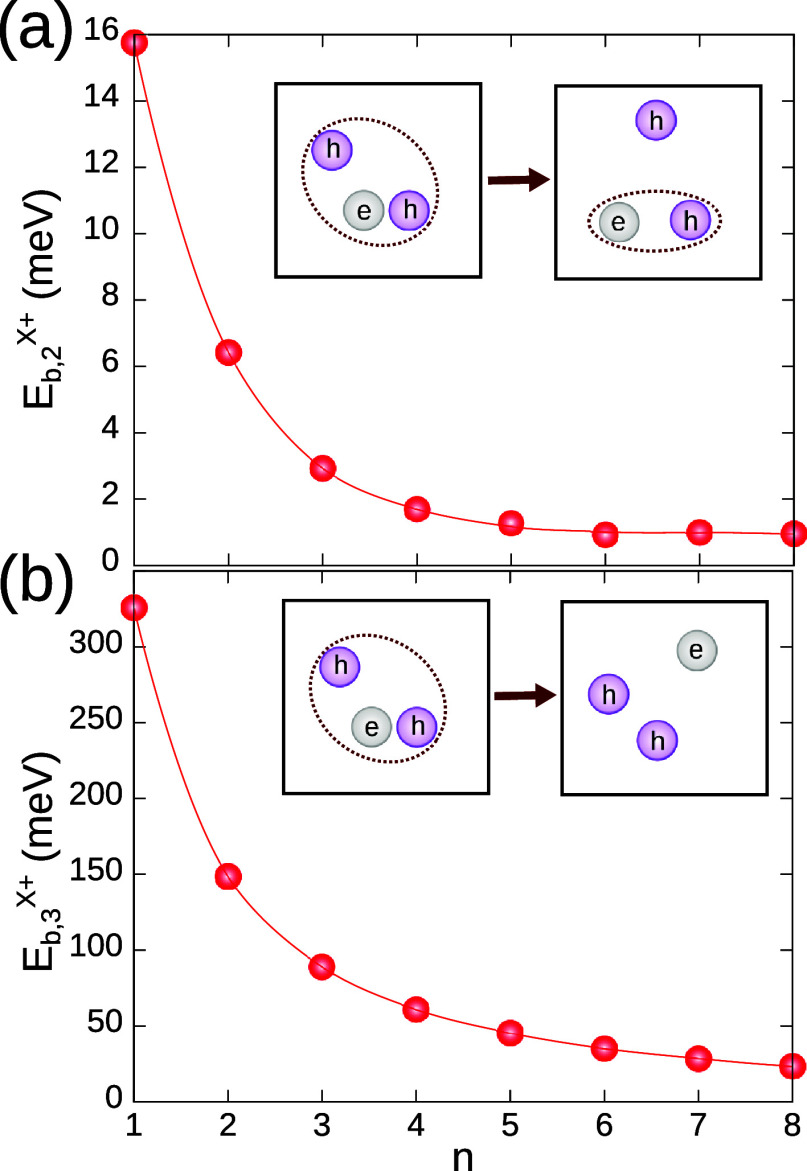
Trion binding energy for the dissociation into two (a) and three (b) uncorrelated particles, as defined in eqs 16 and 17. The insets show the dissociation process under study. The fact that *E*_*b*,3_ ≫ *E*_*b*,2_ implies that most of the correlation energy lies within the exciton. For any LHP thickness, *E*_*b*,2_ is below thermal energy at room temperature, which means that the trion will be formed by an exciton plus a hole, with no mutual correlation.

Correlation binding energies are a measure of the wave function deformation to stimulate Coulomb attractions over repulsions. In rigid quasi-2D nanostructures (i.e., with no Yukawa term favoring ionic structures), it has been shown that trion correlations reduce electron–hole overlap as compared to excitons,^22^ which translates into lower oscillator strength.^23^ Then, a smaller reduction of the oscillator strength is expected in LHPs.

## Conclusions

Ruddlesden–Popper LHP lattices have different polarizability at short and long distances. We have shown that, in thin LHPs, this gives trions a pronounced ionic (asymmetric) character, with the extra carrier orbiting around a closely bound exciton. This configuration optimizes attractions over repulsions, enabling spectroscopic binding energies up to 70 meV, which could exceed those of biexcitons. Correlation binding energies are however smaller. In *X* ^+^, the correlation between a confined exciton and hole is under 16 meV. This means at room temperature the correlation is lost, which should have a direct impact on the radiative rates.

## Supplementary Material



## Notes

Two codes, LocalKinetic.f90 and LocalPotential.f90, corresponding to the Fortran functions employed to calculate the local energy along the random walk of the variational Quantum Monte Carlo method (PDF)
