# Supplementary material for: Electronic Structure of Trions in Layered Hybrid Lead Halide Perovskites
Source: J Phys Chem C Nanomater Interfaces. 2024 Oct 8;128(41):17563–71. doi: 10.1021/acs.jpcc.4c04617 (PMC12315118; doi:10.1021/acs.jpcc.4c04617)
Supplement: Supplementary file 1 [file jp4c04617_si_001.pdf]

Supporting Information for "Electronic Structure of Trions in Layered Hybrid Lead Halide Perovskites"

Juan I. Climente,[†] José L. Movilla,[‡] and Josep Planelles[*,†]

[†]*Departament de Química Física i Analítica, Universitat Jaume I, E-12080, Castelló de la Plana, Spain*

[‡]*Dept. d'Educació i Didàctiques Específiques, Universitat Jaume I, 12080, Castelló, Spain*

E-mail: josep.planelles@uji.es

```fortran
!!!!!!!!!!!!!!!!!!!!!!!!!!!!!!!!!!!!!!!!!!!!!!!!!!!!!!!!!!!!!!!!!!!!!!!!!!!!!!!!
!                                ROUTINE LocalKinetic.f90                       !
!!!!!!!!!!!!!!!!!!!!!!!!!!!!!!!!!!!!!!!!!!!!!!!!!!!!!!!!!!!!!!!!!!!!!!!!!!!!!!!!

    ! Trion local kinetic energy  associated to the Jastrow factor
    ! Exp[-Z(r1+r2)/2] Cosh[Z Q(r1-r2)/2] Exp[Z b r12/(1+Z a r12)]
        !
    ! Kinetic energy is obtained from logarithm of Psi (as in PRB 42, 3503 (1990))
        !
        ! function arguments:
        !        Cartesian coordinates: x1,x2,xe,y1,y2,ye,z1,z2,ze
        !        electron-hole1, electron-hole2 and hole1-hole2 distances: r1,r2,r12
        !        vector dades including:  platelet dimensions Lx,Ly,Lz,kx,ky,kz (ki = pi/
Li)
        !                                 electron and hole masses  mep,mez,mhp,mhz
        !                                 parameters Z, Q, bet, alp

        double precision function Cin(dades,x1,x2,xe,y1,y2,ye,z1,z2,ze,r1,r2,r12)

    Implicit none
        double precision:: x1,x2,xe,y1,y2,ye,z1,z2,ze,r1,r2,r12
    double precision::  Z,Q,bet,alp
    double precision :: Lx,Ly,Lz,kx,ky,kz,mep,mez,mhp,mhz
    double precision :: Fh1x,Fh1y,Fh2x,Fh2y,Fex,Fey
    double precision :: Th1x,Th1y,Th2x,Th2y,Tex,Tey
    double precision :: Fh1z,Fh2z,Fez,Th1z,Th2z,Tez
        double precision, dimension(56) :: dades

    !Retrieve input data from main
    Lx=dades(1)
    Ly=dades(2)
    Lz=dades(3)
    kx=dades(4)
    ky=dades(5)
    kz=dades(6)
    mep=dades(7)
    mez=dades(8)
    mhp=dades(9)
    mhz=dades(10)

    !variational parameters
    Z=dades(19)
    Q=dades(20)
    bet=dades(21)
    alp=dades(22)

!       Kinetic energy obtained from logarithm of Psi (PRB 42, 3503 (1990))


        Fh1x = -((kx*dtan(kx*x1))/(dsqrt(2.d0)*dsqrt(mhp)))+(((-x1+xe)*Z)/(2.d0*r1)-(alp*
bet*(x1-x2)*Z**2)/(1+alp*r12*Z)**2+ &
       (bet*(x1-x2)*Z)/(r12*(1+alp*r12*Z))-(Q*(-x1+xe)*Z*dtanh((Q*(r1-r2)*Z)/2.d0))/(2.d0*
r1))/(dsqrt(2.d0)*dsqrt(mhp))

        Th1x = kx**2/(4.d0*mhp*(dcos(kx*x1))**2)-(-((1/r1-(-x1+xe)**2/r1**3)*Z)/2.d0+bet*Z
*((-2*alp*(x1-x2)**2*Z)/(r12**2*(1+alp*r12*Z)**2)+ &
     (1/r12-(x1-x2)**2/r12**3)/(1+alp*r12*Z)+r12*((2*alp**2*(x1-x2)**2*Z**2)/(r12**2*(1+a-
lp*r12*Z)**3)-(alp*Z)/(r12*(1+alp*r12*Z)**2)+ &
         (alp*(x1-x2)**2*Z)/(r12**3*(1+alp*r12*Z)**2)))+(Q**2*(-x1+xe)**2*Z**2)/(4.d0*r1**
2*(dcosh((Q*(r1-r2)*Z)/2.d0))**2)+ &
         (Q*Z*dtanh((Q*(r1-r2)*Z)/2.d0))/(2.d0*r1)-(Q*(-x1+xe)**2*Z*dtanh((Q*(r1-r2)*Z)/2.
d0))/(2.d0*r1**3))/(4.d0*mhp)

        Fh2x = -((kx*dtan(kx*x2))/(dsqrt(2.d0)*dsqrt(mhp)))+(((-x2+xe)*Z)/(2.d0*r2)+(alp*
bet*(x1-x2)*Z**2)/(1+alp*r12*Z)**2- &
        (bet*(x1-x2)*Z)/(r12*(1+alp*r12*Z))+(Q*(-x2+xe)*Z*dtanh((Q*(r1-r2)*Z)/2.d0))/(2.d0
*r2))/(dsqrt(2.d0)*dsqrt(mhp))

        Th2x = kx**2/(4.d0*mhp*(dcos(kx*x2))**2)-(-((1/r2-(-x2+xe)**2/r2**3)*Z)/2.d0+bet*Z
```

```
*((-2*alp*(x1-x2)**2*Z)/(r12**2*(1+alp*r12*Z)**2)+ &
       (1/r12-(x1-x2)**2/r12**3)/(1+alp*r12*Z)+r12*((2*alp**2*(x1-x2)**2*Z**2)/(r12**2*(1+
alp*r12*Z)**3)-(alp*Z)/(r12*(1+alp*r12*Z)**2)+ &
           (alp*(x1-x2)**2*Z)/(r12**3*(1+alp*r12*Z)**2)))+(Q**2*(-x2+xe)**2*Z**2)/(4.d0*r2
**2*(dcosh((Q*(r1-r2)*Z)/2.d0))**2)- &
           (Q*Z*dtanh((Q*(r1-r2)*Z)/2.d0))/(2.d0*r2)+(Q*(-x2+xe)**2*Z*dtanh((Q*(r1-r2)*Z)/
2.d0))/(2.d0*r2**3))/(4.d0*mhp)

        Fex = -((kx*dtan(kx*xe))/(dsqrt(2.d0)*dsqrt(mep)))+(-(((-x1+xe)/r1+(-x2+xe)/r2)*Z)
/2.d0+(Q*((-x1+xe)/r1- &
      (-x2+xe)/r2)*Z*dtanh((Q*(r1-r2)*Z)/2.d0))/2.d0)/(dsqrt(2.d0)*dsqrt(mep))

        Tex = kx**2/(4.d0*mep*(dcos(kx*xe))**2)-(-((1/r1+1/r2-(-x1+xe)**2/r1**3-(-x2+xe)**
2/r2**3)*Z)/2.d0+ &
      (Q**2*((-x1+xe)/r1-(-x2+xe)/r2)**2*Z**2)/(4.d0*(dcosh((Q*(r1-r2)*Z)/2.d0))**2)+(Q*(1
/r1-1/r2-(-x1+xe)**2/r1**3+ &
          (-x2+xe)**2/r2**3)*Z*dtanh((Q*(r1-r2)*Z)/2.d0))/2.d0)/(4.d0*mep)

        Fh1y = -((ky*dtan(ky*y1))/(dsqrt(2.d0)*dsqrt(mhp)))+(((-y1+ye)*Z)/(2.d0*r1)-(alp*
bet*(y1-y2)*Z**2)/(1+alp*r12*Z)**2+ &
        (bet*(y1-y2)*Z)/(r12*(1+alp*r12*Z))-(Q*(-y1+ye)*Z*dtanh((Q*(r1-r2)*Z)/2.d0))/(2.d0
*r1))/(dsqrt(2.d0)*dsqrt(mhp))

        Th1y = ky**2/(4.d0*mhp*(dcos(ky*y1))**2)-(-((1/r1-(-y1+ye)**2/r1**3)*Z)/2.d0+bet*Z
*((-2*alp*(y1-y2)**2*Z)/(r12**2*(1+alp*r12*Z)**2)+ &
       (1/r12-(y1-y2)**2/r12**3)/(1+alp*r12*Z)+r12*((2*alp**2*(y1-y2)**2*Z**2)/(r12**2*(1+
alp*r12*Z)**3)-(alp*Z)/(r12*(1+alp*r12*Z)**2)+&
           (alp*(y1-y2)**2*Z)/(r12**3*(1+alp*r12*Z)**2)))+(Q**2*(-y1+ye)**2*Z**2)/(4.d0*r1
**2*(dcosh((Q*(r1-r2)*Z)/2.d0))**2)+&
           (Q*Z*dtanh((Q*(r1-r2)*Z)/2.d0))/(2.d0*r1)-(Q*(-y1+ye)**2*Z*dtanh((Q*(r1-r2)*Z)/
2.d0))/(2.d0*r1**3))/(4.d0*mhp)

        Fh2y = -((ky*dtan(ky*y2))/(dsqrt(2.d0)*dsqrt(mhp)))+(((-y2+ye)*Z)/(2.d0*r2)+(alp*
bet*(y1-y2)*Z**2)/(1+alp*r12*Z)**2- &
       (bet*(y1-y2)*Z)/(r12*(1+alp*r12*Z))+(Q*(-y2+ye)*Z*dtanh((Q*(r1-r2)*Z)/2.d0))/(2.d0*
r2))/(dsqrt(2.d0)*dsqrt(mhp))

        Th2y = ky**2/(4.d0*mhp*(dcos(ky*y2))**2)-(-((1/r2-(-y2+ye)**2/r2**3)*Z)/2.d0+bet*Z
*((-2*alp*(y1-y2)**2*Z)/(r12**2*(1+alp*r12*Z)**2)+ &
       (1/r12-(y1-y2)**2/r12**3)/(1+alp*r12*Z)+r12*((2*alp**2*(y1-y2)**2*Z**2)/(r12**2*(1+
alp*r12*Z)**3)-(alp*Z)/(r12*(1+alp*r12*Z)**2)+ &
           (alp*(y1-y2)**2*Z)/(r12**3*(1+alp*r12*Z)**2)))+(Q**2*(-y2+ye)**2*Z**2)/(4.d0*r2
**2*(dcosh((Q*(r1-r2)*Z)/2.d0))**2)- &
           (Q*Z*dtanh((Q*(r1-r2)*Z)/2.d0))/(2.d0*r2)+(Q*(-y2+ye)**2*Z*dtanh((Q*(r1-r2)*Z)/
2.d0))/(2.d0*r2**3))/(4.d0*mhp)

        Fey = -((ky*dtan(ky*ye))/(dsqrt(2.d0)*dsqrt(mep)))+(-(((-y1+ye)/r1+(-y2+ye)/r2)*Z)
/2.d0+ &
       (Q*((-y1+ye)/r1-(-y2+ye)/r2)*Z*dtanh((Q*(r1-r2)*Z)/2.d0))/2.d0)/(dsqrt(2.d0)*dsqrt(
mep))

        Tey = ky**2/(4.d0*mep*(dcos(ky*ye))**2)-(-((1/r1+1/r2-(-y1+ye)**2/r1**3-(-y2+ye)**
2/r2**3)*Z)/2.d0+ &
      (Q**2*((-y1+ye)/r1-(-y2+ye)/r2)**2*Z**2)/(4.d0*(dcosh((Q*(r1-r2)*Z)/2.d0))**2)+ &
          (Q*(1/r1-1/r2-(-y1+ye)**2/r1**3+(-y2+ye)**2/r2**3)*Z*dtanh((Q*(r1-r2)*Z)/2.d0))/
2.d0)/(4.d0*mep)

        Fh1z = -((kz*dtan(kz*z1))/(dsqrt(2.d0)*dsqrt(mhz)))

        Th1z = kz**2/(4.d0*mhz*(dcos(kz*z1))**2)

        Fh2z = -((kz*dtan(kz*z2))/(dsqrt(2.d0)*dsqrt(mhz)))

        Th2z = kz**2/(4.d0*mhz*(dcos(kz*z2))**2)

        Fez = -((kz*dtan(kz*ze))/(dsqrt(2.d0)*dsqrt(mez)))

        Tez = kz**2/(4.d0*mez*(dcos(kz*ze))**2)
```

```
    Cin= 2*Th1x-Fh1x**2+2*Th2x-Fh2x**2+2*Tex-Fex**2 + 2*Th1y-Fh1y**2+2*Th2y-Fh2y**2+2*Tey-
Fey**2+ 2*Th1z-Fh1z**2+2*Th2z-Fh2z**2+2*Tez-Fez**2

    return
    end
```

```fortran
!!!!!!!!!!!!!!!!!!!!!!!!!!!!!!!!
!  ROUTINE LocalPotential.f90  !
!!!!!!!!!!!!!!!!!!!!!!!!!!!!!!!!

DOUBLE PRECISION FUNCTION Coul(dades,x1,x2,xe,y1,y2,ye,z1,z2,ze)
IMPLICIT NONE
DOUBLE PRECISION:: xe,x1,x2,ye,y1,y2,ze,z1,z2
DOUBLE PRECISION:: r1e,r2e,r12,x1e2,x2e2,y1e2,y2e2
DOUBLE PRECISION:: Lx,Ly,Lz
DOUBLE PRECISION:: q,eps1,eps2
DOUBLE PRECISION:: selfe,self1,self2,coule1,coule2,coul12
DOUBLE PRECISION:: Z,alpha,beta, kx,ky,kz,mez,mep,mhp,mhz,mup,muz
DOUBLE PRECISION:: potencial,r3D12,r3D1e,r3D2e
INTEGER :: i
DOUBLE PRECISION, dimension(56) :: dades
!!!!!!!!!!!!!!!!!!!!!!!!!!
DOUBLE PRECISION:: eqw,eps1inf,lpole,lpolh,qY,coul1Y,coul2Y,coul12Y
INTEGER:: diel,yuk
!!!!!!!!!!!!!!!!!!!!!!!!!!

! Retrieve input data from main

Lx=dades(1)
Ly=dades(2)
Lz=dades(3)
kx=dades(4)
ky=dades(5)
kz=dades(6)
mep=dades(7)
mez=dades(8)
mhp=dades(9)
mhz=dades(10)
mup=dades(11)
muz=dades(12)
eps1=dades(17)
eps2=dades(18)


!!!!!!!!!!!!!!!!!!!!!!!!!!!!!!
eps1inf=dades(51)
lpole=dades(52)
lpolh=dades(53)
eqw=dades(54)
diel=dades(55)
yuk=dades(56)
!!!!!!!!!!!!!!!!!!!!!!!!!!!!!!


! Coulomb energy (including dielectric mismatch of quantum well, PRB 40, 12359 (1989))

q=(eps1-eps2)/(eps1+eps2)
coule1=0.d0 ! e-h1
coule2=0.d0 ! e-h2
coul12=0.d0 ! h1-h2
qY=(eqw-eps2)/(eqw+eps2)
coul1Y=0.d0 ! e-h1 yukawa
coul2Y=0.d0 ! e-h2 yukawa
coul12Y=0.d0! h1-h2 yukawa

!    Direct terms
coule1 = coule1+1/(eps1*dsqrt((xe-x1)**2+(ye-y1)**2+(ze-z1)**2))
coule2 = coule2+1/(eps1*dsqrt((xe-x2)**2+(ye-y2)**2+(ze-z2)**2))
coul12 = coul12+1/(eps1*dsqrt((x1-x2)**2+(y1-y2)**2+(z1-z2)**2))
IF(yuk.eq.1) THEN
   coul1Y = coul1Y+0.5d0*(dexp(-dsqrt((xe-x1)**2+(ye-y1)**2+(ze-z1)**2)/lpole)+ &
                          dexp(-dsqrt((xe-x1)**2+(ye-y1)**2+(ze-z1)**2)/lpolh))/ &
                   (eqw*dsqrt((xe-x1)**2+(ye-y1)**2+(ze-z1)**2))
   coul2Y = coul2Y+0.5d0*(dexp(-dsqrt((xe-x2)**2+(ye-y2)**2+(ze-z2)**2)/lpole)+ &
                          dexp(-dsqrt((xe-x2)**2+(ye-y2)**2+(ze-z2)**2)/lpolh))/ &
```

```
                   (eqw*dsqrt((xe-x2)**2+(ye-y2)**2+(ze-z2)**2))
   coul12Y = coul12Y+dexp(-dsqrt((x1-x2)**2+(y1-y2)**2+(z1-z2)**2)/lpolh)/ &
                   (eqw*dsqrt((x1-x2)**2+(y1-y2)**2+(z1-z2)**2))
ENDIF

!    Image terms
IF(diel.eq.1) THEN

! mirror images on one side of well
DO i=-50,-1,1
   coule1 = coule1+q**abs(i)/(eps1*dsqrt((xe-x1)**2+(ye-y1)**2+(ze-z1*(-1)**i - i*Lz)**2))
   coule2 = coule2+q**abs(i)/(eps1*dsqrt((xe-x2)**2+(ye-y2)**2+(ze-z2*(-1)**i - i*Lz)**2))
   coul12 = coul12+q**abs(i)/(eps1*dsqrt((x1-x2)**2+(y1-y2)**2+(z1-z2*(-1)**i - i*Lz)**2))
ENDDO

! mirror images on other side of well
DO i=1,50,1
   coule1 = coule1+q**abs(i)/(eps1*dsqrt((xe-x1)**2+(ye-y1)**2+(ze-z1*(-1)**i - i*Lz)**2))
   coule2 = coule2+q**abs(i)/(eps1*dsqrt((xe-x2)**2+(ye-y2)**2+(ze-z2*(-1)**i - i*Lz)**2))
   coul12 = coul12+q**abs(i)/(eps1*dsqrt((x1-x2)**2+(y1-y2)**2+(z1-z2*(-1)**i - i*Lz)**2))
ENDDO

IF(yuk.eq.1) THEN

! mirror images on one side of well
DO i=-50,-1,1
   coul1Y = coul1Y+qY**abs(i)*0.5d0*(dexp(-dsqrt((xe-x1)**2+(ye-y1)**2+(ze-z1*(-1)**i - i*
Lz)**2)/lpole) + &
                                    dexp(-dsqrt((xe-x1)**2+(ye-y1)**2+(ze-z1*(-1)**i - i*
Lz)**2)/lpolh))/ &
            (eqw*dsqrt((xe-x1)**2+(ye-y1)**2+(ze-z1*(-1)**i - i*Lz)**2))
   coul2Y = coul2Y+qY**abs(i)*0.5d0*(dexp(-dsqrt((xe-x2)**2+(ye-y2)**2+(ze-z2*(-1)**i - i*
Lz)**2)/lpole) + &
                                    dexp(-dsqrt((xe-x2)**2+(ye-y2)**2+(ze-z2*(-1)**i - i*
Lz)**2)/lpolh))/ &
            (eqw*dsqrt((xe-x2)**2+(ye-y2)**2+(ze-z2*(-1)**i - i*Lz)**2))
   coul12Y = coul12Y+qY**abs(i)*dexp(-dsqrt((x1-x2)**2+(y1-y2)**2+(z1-z2*(-1)**i - i*Lz)**
2)/lpolh)/ &
            (eqw*dsqrt((x1-x2)**2+(y1-y2)**2+(z1-z2*(-1)**i - i*Lz)**2))
ENDDO

! mirror images on other side of well
DO i=1,50,1
   coul1Y = coul1Y+qY**abs(i)*0.5d0*(dexp(-dsqrt((xe-x1)**2+(ye-y1)**2+(ze-z1*(-1)**i - i*
Lz)**2)/lpole)+ &
                                    dexp(-dsqrt((xe-x1)**2+(ye-y1)**2+(ze-z1*(-1)**i - i*
Lz)**2)/lpolh))/ &
            (eqw*dsqrt((xe-x1)**2+(ye-y1)**2+(ze-z1*(-1)**i - i*Lz)**2))
   coul2Y = coul2Y+qY**abs(i)*0.5d0*(dexp(-dsqrt((xe-x2)**2+(ye-y2)**2+(ze-z2*(-1)**i - i*
Lz)**2)/lpole)+ &
                                    dexp(-dsqrt((xe-x2)**2+(ye-y2)**2+(ze-z2*(-1)**i - i*
Lz)**2)/lpolh))/ &
            (eqw*dsqrt((xe-x2)**2+(ye-y2)**2+(ze-z2*(-1)**i - i*Lz)**2))
   coul12Y = coul12Y+qY**abs(i)*dexp(-dsqrt((x1-x2)**2+(y1-y2)**2+(z1-z2*(-1)**i - i*Lz)**
2)/lpolh)/ &
            (eqw*dsqrt((x1-x2)**2+(y1-y2)**2+(z1-z2*(-1)**i - i*Lz)**2))
ENDDO

ENDIF

ENDIF

! attractions are negative
coule1=-coule1
coule2=-coule2
coul1Y=-coul1Y
coul2Y=-coul2Y

! E_L : Sum of all terms (self-energy apart)
```

```
!ENE = cinetica+coule1+coule2+coul12+coul1Y+coul2Y+coul12Y
Coul = coule1+coule2+coul12+coul1Y+coul2Y+coul12Y

RETURN
END
```
